# Will It Cricket? Product Development and Evaluation of Cricket (*Acheta domesticus*) Powder Replacement in Sausage, Pasta, and Brownies

**DOI:** 10.3390/foods11193128

**Published:** 2022-10-08

**Authors:** Isaac Ho, Adelynn Peterson, Jack Madden, Evan Huang, Samir Amin, Amy Lammert

**Affiliations:** FSN Department, California Polytechnic State University, San Luis Obispo, CA 93407, USA

**Keywords:** insect-based foods, novel foods, product development, cricket powder, meat products, pasta, brownies

## Abstract

Insect powders used in food products may lower the overall quality when compared to conventional counterparts. This preliminary study was used to develop and evaluate insect-based food products and to utilize them in a future consumer test. Pork sausage, dried pasta, and chocolate brownie formulations were developed to either contain NO cricket powder (Control) or have cricket powder (CP). The products were evaluated for proximate composition and product-dependent parameters. The protein content increased in the CP pasta and brownies (*p* < 0.05) while no changes were found in the sausage (*p* > 0.05). Fat content increased in both the CP pasta and brownies while it decreased in the CP sausage (*p* < 0.05). The CP sausage had a higher carbohydrate content than the Control (*p* < 0.05). Overall, this may be attributed to cricket powder being high in protein and fat while also containing dietary fiber. Cricket powder replacement may lead to noticeable color differences by increasing green and blue coloring in sausage and pasta (*p* < 0.05). Changes in textural properties (*p* < 0.05) may be attributed to cricket powder affecting protein solubility and emulsion stability in sausage while gluten formation may be interfered with in the brownies. Overall, cricket powder replacement had improved nutritional content with minor changes in quality parameters.

## 1. Introduction

By 2050, the human population is expected to reach nine billion [1]. For this reason, food insecurity is an immediate current concern as a proportion of the world population continues to have limited access to nutrient-dense food sources [2]. The United Nations has predicted that food production will need to double to meet the needs of the growing population [3]. Another concern is that livestock production is also expected to double between 2000 and 2050 as developing countries adopt animal proteins as a primary food source [4,5]. Livestock farming is detrimental to the environment due to its overconsumption of resources, such as land and water, as well as its contribution to overall greenhouse gas emissions [4,6]. As a result, the development of sustainable food sources may be necessary to mitigate the effects of livestock production while also providing food security.

Insects as an edible food source may be a promising solution. Insects have a high protein content and can provide all essential amino acids necessary for the human diet [7,8,9]. When comparing insect rearing to traditional livestock farming, insects require fewer resources such as lower feed requirements than their livestock counterparts [1,6]. Not to mention, insect farming may be more efficient than livestock farming as land usage can be optimized through vertical farming [1,6,10,11]. Currently, agricultural land in South America and sub-Saharan Africa has been expanded to meet livestock demand, leading to deforestation [2]. As a result, insect farming may reduce land usage while supplying the demands for protein sources. 

Another consideration in the future may be which insect species to farm may need to farm as the nutritional profiles between species may vary [8,12]. In Asian countries, such as China, insect consumption has been engraved into the history of certain regions [13,14,15]. In China, 34 edible insects, which includes silkworm larvae (*Bombyx mori*), mealworm larvae (*Tenebrio molitor*), and Orthoptera species (locusts, grasshoppers, and mole crickets), have been developed and assessed for production as food sources or for medicinal purposes [15]. 

However, house crickets (*Acheta domesticus*) and mealworm larvae (*Tenebrio molitor*) may be the most viable for the Western market. Western government bodies have recently deemed these insect species as safe for human consumption as long as they are produced in safe-rearing conditions [16,17,18]. There has been a focus on these insect species in sensory and consumer research as well as startup companies developing insect farming for industrial-size production [6,19]. For powders, companies such as EXO, Griopro^®^, JR Unique Foods, and Entomo Farms have made cricket powder commercially available in the Western market [20,21]. Other companies already offer other products containing crickets or mealworms such as protein bars (EXO, Landish, Kriket, and Jimini’s), pasta (Kric8, Hoppa Foods, and Bugsolutely), and snack products such as chips and baked goods (Chirps, Don Bugito, and Circle Harvest) [20,21]. By 2023, the market for insect-based food products is projected to reach 50 million USD [22]. Therefore, insects may offer more opportunities for the food industry and provide accessible alternative protein sources for consumers.

However, consumers may be deterred from consuming insects as a food source due to psychological factors. Disgust, a culturally induced form of rejection, is a way for humans to avoid foods that may lead to illness and disease [23,24]. In Western countries, consumers have an association of insects with filthiness and contamination [24,25,26]. Studies have found that disgust can negatively correlate to Western consumers’ willingness to consume insects [18,19,25,27]. Not to mention, other factors such as food neophobia or familiarity with entomophagy may play another role in the acceptance of insects [28]. 

Though, the acceptance of insects may be more common in countries that have a history of consuming insects. For example, Verneau et al. [29] found that Chinese consumers who have previously consumed insects self-reported less disgust with more intent to consume them than those who have not eaten insects. In Latin American countries, insect consumption is dependent on the region. Small ethnic groups, with limited Western influence, may be more familiar with entomophagy as insects are already consumed as a food source [30,31]. In Brazil, consumers may still be influenced by negative perceptions due to safety and nutritional concerns [32,33]. However, Brazilian consumers may have more intention to replace conventional meat products with insect-based proteins, or even whole insects if they are more familiar with insect consumption [33,34]. Bisconsin-Júnior et al. [35] also supports this as Brazilian consumers may be more willing to consume insects (crickets, grasshoppers, and ants) if they were either fried or roasted when prepared. To increase the familiarity of entomophagy in the Western market, developing methods to understand and reduce the factors that may negatively affect their acceptance may be necessary.

Research suggests that reducing the disgust factor while also increasing interest to consume insects may encourage consumers to try insects. Studies have found that consumers in Western countries may be more willing to try insects if they are in nonvisible forms (powders and ground meals) than visible forms (whole insects) [18,36,37,38]. Not to mention, interest plays a key role that may deter the negative effects of disgust towards insects. Serpico et al. [39] suggested that reducing the negative emotions associated with insects and increasing positive emotions, such as interest and curiosity, may increase the acceptance of insects in food. Placentino et al. [40] found when conducting a study with Italian athletes that protein content and the curiosity of texture were the main drivers to taste a protein bar containing cricket flour. To develop familiar products containing insects, perceived food appropriateness may need to be considered as unfamiliar food products, such as ethnic cuisines, may further deter consumers [18,41,42,43]. Insects used in unfamiliar food products, such as ethnic cuisines, may further deter Western consumers from accepting them [18]. As a result, when developing insect-based food products for the Western market, interest in consuming insect-based food products may coincide with food appropriateness and familiarity.

One issue when incorporating insects into familiar conventional food products may be consumers’ expectations regarding the sensory characteristics and quality of the product [42,44,45]. The addition of insects in the product may change those characteristics and qualities [41,42]. A study evaluating the consumer acceptance of brownies with cricket powder rated brownies containing cricket powder lower in consumer acceptability than brownies that did not contain cricket powder [46]. Another study also found that increasing cricket powder in crackers can change the textural properties as well as increasing rejection by consumers [47]. When introducing insects into food products, the amount of insect used may need to be considered in food formulations. This may ensure that insect-based products may be as acceptable as other competitive products already available in the market. 

For this study, the three different food products (sausage, pasta, and chocolate brownies) were developed to either contain cricket (*Acheta domesticus*) powder (CP) and NO cricket powder (Control). This study was conducted to be a preliminary study to develop three different food products for a larger and more complex study involving consumer testing. The products were selected to be used as the main components for a three-course meal (appetizer, entrée, and dessert) to predict the consumer acceptability of insect-based food products. These products were selected as previous research has mainly focused on incorporating insects into snack foods (chips, crackers, cookies, etc.) [18]. Snack foods can be a readily available option for consumers [47]. This allows for an easy delivery method for the incorporation of insects [48]. However, there is limited research on the consumer acceptability of complete dishes containing insects. Not to mention, the development of these products allowed for the incorporation of cricket powder into the formulations which may be more acceptable to consumers who prefer nonvisible insects. The specific objectives of this study were to (1) develop insect-based food products that are comparable to conventional food products, (2) to compare the nutritional properties and food quality of the conventional food products with and without the addition of cricket powder, and (3) to utilize the developed products in future consumer testing.

## 2. Materials

**Figure A1:**
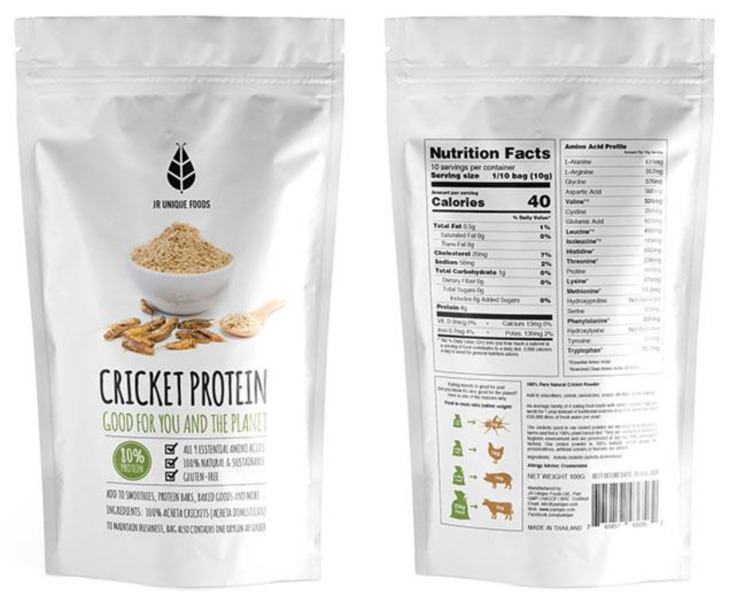
Cricket (*Acheta domesticus*) powder used in the study.

Three different product (sausage, pasta, and chocolate brownies) formulations were developed. A base formulation (Control) for each product was developed, and the cricket powder (CP) versions were developed by replacing a major ingredient component with cricket (*Acheta domesticus*) powder. Appendix A shows the cricket powder used (Figure A1)and its nutritional content (Table A1). Preliminary testing was used to determine the optimum replacement levels in each product. This was conducted by maximizing cricket powder usage without diminishing the sensorial and quality attributes when compared to the Controls. The products were prepared with different replacement levels of cricket powder and evaluating them for the eating quality (taste, flavor, mouthfeel, and texture) as well as product-dependent quality measurements later discussed. Final cricket powder replacement levels were selected and used for the CP versions. The Control and CP formulations used and how they were prepared for testing are discussed below. All ingredients used were purchased from local grocery stores within the San Luis Obispo area.

### 2.1. Sausage

A mild Italian pork sausage was developed using a pork blend of 70% lean meat and 30% pork fat as the base formulation (Control). For the CP formulation, 10% of the lean meat used in the pork blend was replaced with cricket powder. 

A 1000 g batch was made for the Control and CP versions of the pork sausage. Sausage development was performed in two steps: the development of the meat blend and the sausage blend. For the meat blend, a 2:1 ratio of lean meat to pork fat was used to make the 70/30 lean-to-fat sausage blend [49]. From that ratio, 10% of the lean meat used was replaced with cricket powder for the CP sausage. For example, if 100 g of lean meat and 50 g of pork fat were used in the meat blend, 10 g of lean meat would be replaced with cricket powder. Table 1 shows the usage levels of the pork lean, pork fat, and spice mix used in the sausage blend. For the CP pork blend, 10% of the lean meat that was replaced with cricket powder led to a final usage level of 6.23% in the final formulation. 

Pork butt fat was trimmed to be used as the pork fat for the sausage blend. The meat and fat were cut into 2 cm size pieces and were stored in vacuum-packed bags and frozen at −10 °C before grinding. Before grinding, the pork meat and fat were removed from the freezer and were cut into sizes that can fit through the meat grinder. The spice mix was pre-weighed and mixed into the frozen pork pieces before grinding. For the CP version, the cricket powder was also mixed in during this step. 

The pork meat, fat, and spices were ground through a meat grinder (Big Bite Model #8, L.E.M. Products, West Chester, OH, USA). The grinder pieces were chilled in the freezer for at least one hour before grinding. The Control sausage was first made by feeding the pork blend through the meat grinder using a coarse size plate (10 mm). The coarse sausage was then fed through the grinder using a fine size plate (4.5 mm) twice for a total of three grinding steps. Ice was run through the meat grinder to remove residual meat left in the grinder and to chill it before making the CP sausage using the same procedure as the Control. 

The sausage batches were vacuum-packed and frozen at −23.3 °C for storage. The sausage batches were placed in a refrigerator at around 4 °C at least one day before completing any analyses. Analyses were conducted within one week after preparation.

### 2.2. Pasta

Dried fresh fettuccine pasta was developed using a durum wheat-based formulation. For the Control pasta, 14% of the durum wheat semolina was replaced with whole wheat flour while 5% of the durum wheat semolina was replaced with cricket powder for the CP pasta. Whole wheat flour replacement was selected for the Control pasta as Duda et al. [50] found that pasta containing cricket powder has similar appearances, such as color, to commercially available whole wheat pasta. Pasta samples were evaluated for proximate analysis, cooking properties, and textural properties.

A 500 g batch was made for the Control and CP pasta. Table 2 shows the usage levels for both versions of the pasta formulations. To enhance the color and create similar products in appearance, powdered caramel coloring was added to both formulations at 0.10% usage and 0.075% usage for the Control and CP pasta, respectively. For the Control, more water was needed to hydrate the dry mix compared to the CP pasta formulation. However, this may be negligible as the pasta would be later dried.

The durum wheat flour, whole wheat flour or cricket powder, and caramel coloring were premixed beforehand to ensure homogeneity of the dry ingredients. The dry ingredients were then added to an Avancini pasta machine (Model TR 70, Molina di Malo, Vicenza, Italy). The pasta machine mixed the dry ingredients further then water was slowly added to the mix. The pasta dough was extruded through a bronze fettuccine pasta die (10 mm in width), and the pasta was cut when the pasta reached 30 cm in length. The fresh pasta was hand wrapped into bundles and placed onto a baking sheet lined with parchment paper and dusted with additional durum wheat semolina flour. The fresh pasta was left at room temperature for 24–36 h or until the pasta was completely dried. The dried pasta was packaged in 1-gallon Ziploc (brand, location) resealable bags and stored in a cool, dry environment. Analyses were conducted within one week after production.

### 2.3. Brownies

The brownie formulation used a modified formulation developed by Gisslen [51]. The CP brownie was developed by replacing 7% of the wheat flour with cricket powder. During preliminary testing, 7% cricket powder was found to be the most optimum without altering the appearance and eating quality of the brownie such as aftertaste and texture. 

A 1000 g batch was made for each brownie version. Table 3 shows the usage levels for each ingredient used. The brownies were prepared following the recipe instructions by Gisslen [51]. For the cricket powder, preliminary testing found that it gave the brownies a grainy mouthfeel. To reduce this, the cricket powder was ground further using a grinder (CGOLDENWALL Model CNA 923D, Zhejiang, China) with dry ice (Cuesta Springs Ice Co., San Luis Obispo, CA, USA) for 3 min. 

The bittersweet chocolate, butter, and cocoa powder were first heated in a double boiler to melt and mix the chocolate and butter as well as bloom the cocoa powder. Once completely melted, the mixture was set aside to cool to room temperature. The eggs, sugar, salt, and vanilla extract were then mixed in a separate bowl until uniform. The egg mixture was then poured into the melted chocolate mixture and mixed until uniform. The wheat flour (and cricket powder for the CP version) was sifted in and the batter was folded using a rubber spatula. The chocolate chunks were folded in at this time.

The brownie batter was poured into an ungreased 20.3 cm × 30.5 cm (8″ × 12″) baking pan lined with aluminum foil. The brownies were baked in an oven set at 162.8 °C (325 °F) for 35 min. The brownies were removed from the oven and allowed to cool and equilibrate to room temperature. For proximate analysis, the brownies were wrapped in aluminum foil and plastic wrap and stored in a freezer set at −23.3 °C until testing.

## 3. Methods

### 3.1. Sausage

Sausages were evaluated for proximate analysis, cooking properties, and physicochemical properties [52].

#### 3.1.1. Cooking Yield

Sausage batches were made into 75 g patties and were cooked using an Avantco sandwich grill (Model P70S, Meridian, ID, USA) set at 176.7 °C (350 °F). Patties were cooked for 3 min or until the internal temperature reached a final temperature of 71.1 °C (160 °F) or above for ground meats [53]. The cooked patties were then allowed to cool to room temperature. The initial (raw) and final (cooked) weights of the patties were recorded, and the cooking yield was determined using the following equation:

$$\mathrm{Cooking}\;\mathrm{Yield}\;(\%)=\frac{\mathrm{Weight}\;\mathrm{of}\;\mathrm{Cooked}\;\mathrm{Sausage}\;\mathrm{Patty}}{\mathrm{Weight}\;\mathrm{of}\;\mathrm{Raw}\;\mathrm{Sausage}\;\mathrm{Patty}}\;\times \;100\%$$

The cooking yield procedure was performed in triplicate. Cooked sausage patties were used for further analyses.

#### 3.1.2. Color Measurements

The raw and cooked sausage were analyzed for color to determine noticeable color differences between the Control and CP sausage. A FRU^®^ Precise Color Reader (Model WR-18, Shenzhen Wave Optoelectronics Technology Co., Ltd., Shenzhen, China) was placed directly onto the raw sausage to analyze color. Color measurements for the raw sausage were performed immediately after grinding to limit any oxidation of the meat that may have occurred. For the cooked sausage, the patties were sliced, and the colorimeter was directly placed onto the cross-sections. This procedure was performed in triplicate. Color parameters were represented as color variables L* (a higher value means lighter in color while lower means darker in color), a* (a higher value means redder in color while lower means greener in color), and b* (a higher value means bluer in color while lower means yellower in color). Color difference (ΔE) was calculated using the mean of the color values in the following equation:

ΔE_Lab_ = √[(ΔL*)^2^ + (Δa*)^2^ + (Δb*)^2^]

where ΔE_Lab_ is the mean color difference between Control and CP versions when using the Control as the reference.

As the equation shows, ΔE cannot be lower than 0 and can be interpreted universally by Wojciech and Maciej [54] as the following:

1. 0 < ΔE < 1—the difference is not noticeable;
2. 1 < ΔE < 2—the difference is noticeable only for experienced observers;
3. 2 < ΔE < 3.5—the difference is noticeable also for inexperienced observers;
4. 3.5 < ΔE < 5—clear color difference;
5. 5 < ΔE—observers notice two different colors [55].

#### 3.1.3. pH Measurements

The raw and cooked sausages were evaluated for pH using a modified procedure described by Park et al. [56]. For the raw sausage, 5 g of the raw sausage was mixed with 20 mL of distilled water and blended for 1 min using a homogenizer (Yjingrui Model AD300L-H, Zhengzhou, China). For the cooked sausage, 3 g of the cooked sausage was mixed with 27 mL of distilled and homogenized using a Vitamix blender (Model 5200, Olmstead, OH, USA) by blending the mixture for 60 s. Due to the cooked sausage texture, the Vitamix blender was more efficient than the homogenizer to make a homogeneous mixture as found during preliminary testing. The pH was measured using a Hanna pH meter (Model HI99161, Hanna Instruments, Woonsocket, RI, USA) at 21.6 ± 0.3 °C for the raw sausage and 25.6 ± 0.2 °C for the cooked sausage. This procedure was performed in triplicate.

#### 3.1.4. Textural Properties

Texture profile analysis (TPA) was conducted after the cooked sausage patties, in triplicate, on both sausage versions, were allowed to equilibrate at room temperature. Sausage samples were taken by cutting out a circle from the center portion of the patty using a 35 mm-diameter circle cookie cutter. A Brookfield texture analyzer (Model CT3 10K, Middleboro, MA, USA) was used to evaluate firmness, cohesiveness, and springiness using two cycles of compression. The texture analyzer used the following settings: a TA5 probe attachment (acrylic cylinder with 12.7 mm diameter and 35 mm length), speed set at 2.00 mm/s, and 50% deformation. 

### 3.2. Pasta

Pasta samples were evaluated for proximate analysis, cooking properties, and textural properties.

#### 3.2.1. Cooking Properties

Optimum cooking time, water absorption, and cooking loss were determined according to AACC International Method 66-50.01 [57]. For optimum cooking time (OCT), 25 g portions of the dried pasta samples were boiled in 300 mL of distilled water. Pieces of pasta were removed from the boiling water at 30-s intervals and squeezed between clear plastic by hand. Once the white core of the pasta disappeared, the time would be recorded as the cooking time. This procedure was performed in triplicate.

For water absorption (WA), 25 g portions of dried pasta were weighed and boiled in 300 mL of distilled water to their OCT. Once the OCT was achieved, the cooked pasta was strained using a strainer with a 16 oz plastic deli cup (Choice Foodservice Products, WebstaurantStore, Lancaster, PA, USA) placed underneath to collect the pasta water. An additional 50 mL of distilled water was poured over the strained pasta to rinse the cooked pasta. The rinse water was collected with the pasta water and would later be used to determine cooking loss. The cooked pasta was then weighed, and water absorption was determined using the following equation described by Biró et al. [55]:

$$\mathrm{WA}\;(\%)=\frac{\mathrm{Weight}\;\mathrm{of}\;\mathrm{Cooked}\;\mathrm{Pasta}\;-\mathrm{Weight}\;\mathrm{of}\;\mathrm{Dried}\;\mathrm{Pasta}}{\mathrm{Weight}\;\mathrm{of}\;\mathrm{Dried}\;\mathrm{Pasta}}\;\times \;100\%$$

This procedure was conducted in triplicate. The cooked pasta was used to measure color and textural properties.

For cooking loss, the collected pasta water was evaluated using a modified method described by the AACC Method 66-50.01. Subsamples (aliquots) from each pasta water sample were used as the researchers did not have the equipment to dry the pasta water. The pasta water was pre-weighed and 10 g aliquots were dispensed into pre-weighed crucibles in triplicate. For each pasta version, there were three aliquots for each of the three pasta water samples collected, 18 in total. The crucibles were placed into a Blue M forced-draft air oven (Model ESP-400BC-4, Blue Island, IL, USA) set at 100 °C. The crucibles were left in the oven and weighed periodically over a week until the crucible (with dried solids) weights were constant. The remaining % solids left from each aliquot were determined with the following equation:

$$\%\;\mathrm{Solids}=\frac{a-b}{c}\;\times \;100\%$$

where a is the final crucible weight with remaining solids, b is the crucible weight, and c is the weight of the aliquot. The average % solids from the aliquots were then used to determine cooking loss found in each pasta water using the following equation:

$$\mathrm{Cooking}\;\mathrm{Loss}\;(\%)=\frac{\mathrm{xy}}{z}\;\times \;100\%$$

where x is the average % solids remaining from each aliquot, y is the weight of pasta the aliquots were retrieved from, and z is the weight of the dried pasta the pasta water was collected from after cooking. This average cooking loss was then determined from the three pasta water samples collected during the study.

#### 3.2.2. Color Measurements

Color was measured for both the dried pasta and cooked pasta in triplicate. The dried pasta was measured for color by using a modified procedure described by Švec et al. [58]. For the dried pasta, 10 g portions of dried pasta were ground into a powder by blending them in a Vitamix blender for 1 min. The pasta powder was then sifted using a 500 µm standard sieve to remove any large particles and create a more uniform powder in particle size. The powder was placed into a small, shallow aluminum, and the powder was flattened using an offset spatula to ensure uniformity in color. An FRU^®^ Precise Color Reader was directly placed onto the pasta powder and color was measured. For the cooked pasta, 5 g portions of cooked pasta were wrapped in plastic wrap and mashed using a pestle to flatten the pasta and ensure uniformity in color. The plastic wrap was removed, and the Precise Color Reader was directly placed onto the flattened pasta to measure color.

Differences in color between the dried pasta samples and cooked pasta samples were determined using the procedure described in Section 3.1.2. 

#### 3.2.3. Textural Properties

TPA was conducted using the Brookfield texture analyzer to evaluate firmness, cohesiveness, and springiness. The following settings were used: TA7 probe (acrylic knife edge with 60 mm in width), speed set at 2.00 mm/s, and 50% deformation. The pasta was equilibrated to room temperature before conducting TPA. Four strands of pasta were placed adjacent to each other under the probe to ensure that the probe was in full contact with the pasta when conducting TPA. This procedure was performed in triplicate.

### 3.3. Brownie

The brownies were evaluated for proximate analysis and textural properties.

#### Textural Properties

TPA was conducted using the Brookfield texture analyzer to evaluate firmness, springiness, and chewiness. TPA was conducted the same day the brownie batches were prepared and allowed to equilibrate to room temperature. The following settings were used: an acrylic cylinder probe (TA4, 38.1 mm in diameter, 20 mm in length), speed set at 2.00 mm/s, and 50% deformation. The edges of the brownies were removed by cutting off 1 cm on all sides and the brownies were then cut into 3 cm × 3 cm squares. Only the squares from the center of the brownie were used for TPA. 

### 3.4. Proximate Analysis

Both versions (Control and CP) of the raw sausage, dried pasta, and baked brownie were analyzed for proximate composition on a dry basis [59]. Samples were pre-weighted and placed into an HFS vacuum oven (Model DZF-6050, HFS Inc., Azusa, CA, USA) set at 70 °C for 16 h. The set temperature was selected due to the high-sugar content of the brownies, which may lead to decomposition of the sugars into water, as well as the high-fat content of the sausage, which may lead to oxidation of the fatty acids increasing weight gain in the samples [60]. Samples were removed and weighed, and the loss from drying was used to determine moisture content. The dried samples were used to determine protein content, fat content, and ash. Protein content was determined using the Kjeldahl method (FOSS Tecator™ Digestor, FOSS Kjeltec™ 8200 Auto Distillation Unit, Eden Prairie, MN, USA) to determine the percentage of nitrogen in the sample (AOAC 981.10). A nitrogen-to-protein conversion factor of 5.17 for meat products was used to determine the protein content for the sausage, a conversion factor of 5.7 for wheat flour was used for the pasta, and a conversion factor of 6.25 for standard food products was used for the brownie [61]. Fat content was determined using Soxhlet extraction (FOSS Soxtec™ 2043, Eden Prairie, MN, USA) with petroleum ether (AOAC 991.36). The fat extracted samples were used to conduct ash analysis by placing them in a muffle furnace (Barnstead Thermolyne 62700, Thermolyne Corporation, Dubuque, IA, USA) at 550 °C for 24 h. Total carbohydrates (simple sugars, complex carbohydrates, and dietary fiber) were calculated by difference.

### 3.5. Data Analysis

All measurements were conducted in triplicate. A two-sample t-Test was carried out independently for each dependent variable (parameter measured) at α = 0.05. Statistical analyses were conducted using JMP Pro version 15.1 (SAS Institute, Cary, NC, USA).

## 4. Results & Discussion

### 4.1. Sausage

**Figure 1:**
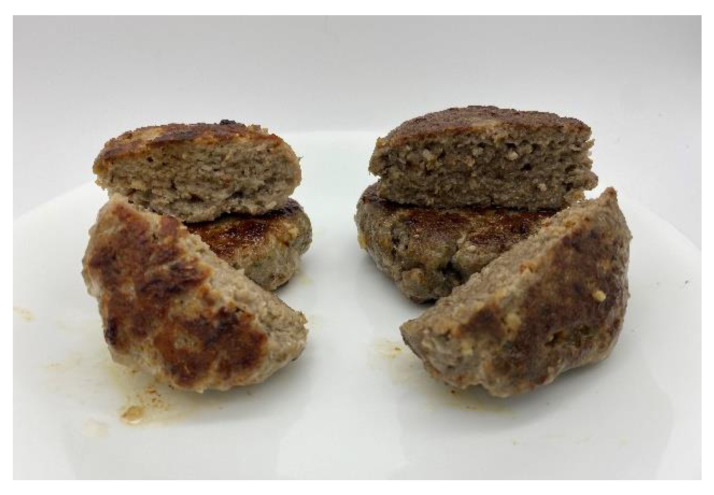
Control (**left**) and CP (**right**) sausage patties.

Figure 1 shows the cooked sausages developed. Table 4 shows the measurements conducted on both sausages.

#### 4.1.1. Sausage Composition

The CP sausage was significantly lower (*p* < 0.05) in moisture and crude fat content (54.88% and 42.52%, respectively) than the Control (59.77% and 48.61%, respectively). There were no differences found for ash and protein between the Control and CP sausages (*p* > 0.05). However, carbohydrate content was significantly higher (*p* < 0.05) for the CP sausage (20.81%) than for the Control (14.20%).

The CP sausage may have had a lower moisture content (*p* < 0.05) than the Control due to the lean meat being replaced with cricket powder, a lower moisture product. However, there was no change in protein content between the two versions (*p* > 0.05). Though, proximate analysis was conducted on the raw sausage. Previous research has found that cooked meat products, that had lean meat and/or fat replaced with edible insect flours, had an increase in protein content [5,52,62]. A decrease in moisture but an increase in protein (*p* < 0.05) may be attributed to the higher solid content found in edible insect flours when compared to animal meat [5,52,63]. 

The CP sausage fat content was significantly lower (*p* < 0.05) than the Control which may be attributed to the cricket powder used; however, the cricket powder contained 6.7 g total fat per 100 g (Appendix A) with the pork shoulder butt reported containing 16 g total fat per 100 g. Replacing the pork shoulder meat, which has higher fat content than cricket powder, may have led to a decrease in fat content [63]. However, other studies evaluating cooked sausages with up to 10% insect flour replacement either found an increase or no change in fat content [5,62]. 

The CP sausage was found to have a higher carbohydrate content (*p* < 0.05) when compared to the Control. The cricket powder used in this study contained 5.5 g of carbohydrates per 100 g (Appendix A). This is relatively lower than other commercial cricket powders which have been found to contain around 19.6–21.8% carbohydrates (dry matter) [64]. By replacing the lean meat, which contains 0 g of carbohydrates from the nutrition facts, with cricket powder, the carbohydrate content may have increased in the CP sausage. The carbohydrate content found in the Control may have been attributed to the spices and herbs used in the formulation. Furthermore, the carbohydrate content found in the CP sausage may be attributed to chitin, a dietary fiber, found in the exoskeleton of insects [52,64]. The dietary fiber content of the cricket powder was 5.4 g per 100 g, approximately 98.1% of the total carbohydrate content (Table A1). Though dietary fiber was not analyzed, the addition of cricket powder may increase fiber content in meat products.

#### 4.1.2. Color

The color of the sausage darkened in appearance with the addition of cricket powder (Figure 1). For both the raw sausage and cooked sausage patties, there was a noticeable difference between the two versions (1 < ΔE < 2). This can be further supported as the raw CP sausage was significantly greener (lower a* value) than the raw Control sausage (*p* < 0.05). For the cooked sausage, the a* and b* values of the CP sausage were significantly lower (*p* < 0.05). This indicates that the cooked CP sausage was higher in green and blue hues than the cooked CP sausage.

Previous studies found that the addition of mealworms (*Tenebrio molitor*) increased the darkness of sausage emulsions with an increase in yellowness [5,63]. The raw sausage had a decrease in lightness (lower L* value), though no difference (*p* > 0.05), and became significantly greener (*p* < 0.05) with the addition of cricket powder. For the cooked sausage, there was no difference in lightness (*p* > 0.05); however, there were more green and blue hues found in the CP sausage than in the Control. Smarzyński et al. [65] also supports this as the addition of cricket powder in pork pâté increased green and blue coloring.

#### 4.1.3. pH, Cooking Yield, and Textural Properties

No differences were found for pH between the Control and CP sausages when either raw or cooked (*p* > 0.05). The CP sausage had a significantly higher (*p* < 0.05) cooking yield (81.08%) than the Control (69.08%). For textural properties, the firmness and cohesiveness of the CP sausage were significantly lower (*p* < 0.05) than the Control. However, the springiness of the CP sausage was found to be significantly higher (*p* < 0.05) than the Control sausage.

The pH can be used as an indicator for the stability of the sausage emulsions. A low pH may cause protein denaturation which may affect color, protein solubility, water holding capacity, and microbial spoilage [66]. However, there was no change in pH with the addition of cricket powder in both the raw and cooked sausage (*p* > 0.05). This may be due to the cricket flour/powder having a similar pH to lean pork meat and pork fat [52]. Protein solubility of emulsified meat products may attribute to the emulsion stability, yield, and texture [52,67]. The protein solubility of cricket protein (*Acheta domesticus* and *Gryllodes sigillatus*) has been found to decrease under acidic conditions due to its isoelectric pH [52,68]. This may be a concern as lean pork meat contains more salt-soluble proteins [52]. By replacing the lean meat portion with cricket powder, the total protein solubility of the sausage may decrease which may affect the texture. Though, protein solubility was not measured in this study. 

The cooking yield was higher in the CP sausage than in the Control (*p* < 0.05). Other studies also found that cooking yield increased (or reduced cooking loss) with up to 10% insect flour/powder replacement [5,52,56]. Kim et al. [63] explained that this may be due to the reduced moisture content and higher solid content in meat emulsion formulations containing insect powders.

For textural properties, the firmness and cohesiveness decreased while springiness increased with cricket powder replacement (*p* < 0.05*).* Powders/flours from different insect species and sourcing, as well as the method of sausage preparation, may alter the textural properties of the meat emulsion to certain degrees. Both Kim et al. [52] and Cruz-López et al. [45] prepared sausages in cellulose casings. Kim et al. [52] found that hardness increased with cricket powder replacement with no changes in cohesiveness and springiness. Cruz-López et al. [62] found that the addition of grasshopper (*Sphenarium purpurascens*) flour increased the hardness, springiness, gumminess, and chewiness. 

The use of cricket powder may be a viable food ingredient in meat emulsions as a meat extender. Meat products are inherently part of many food cultures [69]. To reduce meat consumption, an option may be to partially replace animal meat with other viable sources such as plant proteins, or even insect proteins. This would increase the yield of meat products which may inadvertently decrease animal meat production. 

Meat replacement with plant-based sources can modify meat products to be similar to conventional products [69,70]. The use of insect powders, such as cricket powder, may be a viable source to maintain protein content or even increase it without having negative impacts on the meat product [52] The addition of insect powders in meat products may be beneficial to increase the mineral content and fiber content [5,52]. However, previous research has found that meat replacement of over 10% with insect powders may reduce the quality of the meat product such as emulsion stability [5,62]. To the authors’ knowledge, this study is one of few that evaluates sausage emulsions with cricket powder. 

The purpose of this work was to develop different food products and not to optimize the functionality of cricket powder in the products tested. As a result, additional functional properties such as water holding capacity, other textural properties, and fiber were not analyzed.

### 4.2. Pasta

**Figure 2:**
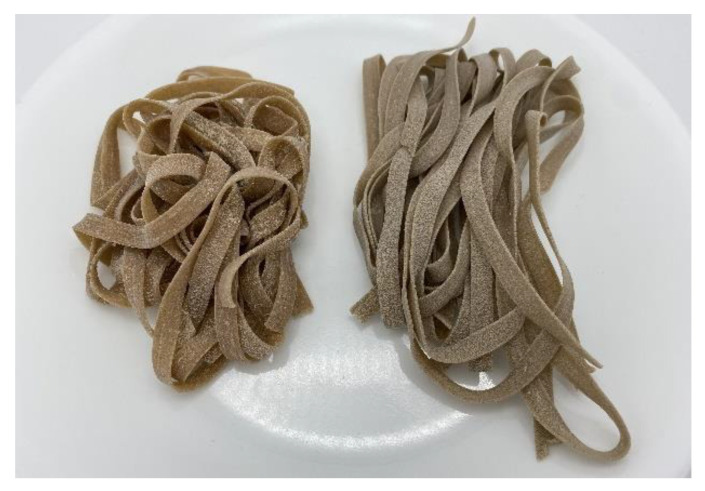
Control (**left**) and CP (**right**) dried pastas.

Figure 2 shows the dried pasta for both the Control and CP versions. The measurements conducted on the pastas are found in Table 5.

#### 4.2.1. Pasta Composition

The protein and fat content for the CP pasta (16.83% and 1.23%, respectively) were significantly higher (*p* < 0.05) than the Control (13.13% and 0.22%, respectively). The carbohydrate content for the CP pasta (77.65%) was found to be significantly lower (*p* < 0.05) than the Control (82.48%). No differences were found in moisture content and ash between the Control and CP pasta (*p* > 0.05).

There was an increase in both protein and fat content between the Control and CP pasta (*p* < 0.05). The increase in protein and fat may be attributed to the replacement of durum wheat with cricket powder as the semolina contained 13.2 g of protein per 100 g compared to the cricket powder which contains 78.0 g of protein per 100 g (Appendix A). Commercial cricket powder has been found to contain 42.0–65.5% in protein content and 16.1–29.1% in fat (dry matter) [64,71]. This can be further supported as cricket powder replacement, at levels of 5% or higher, in other pasta formulations, were found to have an increase in protein and fat content [50,72,73]. The low fat content of the Control pasta may be due to the durum wheat flour as most of the lipids in wheat come from the germ, which is removed during processing [74]. 

For wheat-based staple foods, such as pasta, the addition of insect powders may be a viable option to increase the nutritional profile. The CP pasta had a decrease in carbohydrate content but was found to have an increase in protein and fat when compared to the Control (*p* < 0.05). Pasta is known for its low glycemic index and can be further reduced with higher protein content [75]. Pasta enriched with plant-based proteins is already available in the market; however, the concern with plant proteins may be their low digestibility compared to animal proteins [72,76]. Insect proteins have a higher digestibility than plant proteins with a range of 76–96% of absorption in the human body [11,72,77]. The durum wheat used in the study contained 13.1% protein (wet basis); however, cereal grains such as durum wheat semolina lack valuable amino acids such as lysine and tryptophan [78]. Previous studies have found that the addition of cricket powder in pasta has been found to improve the amino acid composition [50,79,80]. As a result, insect-based pasta may be an option for consumer groups who may be looking for staple foods that contain all essential amino acids for the human diet.

#### 4.2.2. Pasta Color

The difference in color between the powdered dried pasta was not noticeable (0 < ΔE < 1). This can be further supported as there were no differences found between the color values for the dried pasta (*p* > 0.05). However, when cooked, there was a clear difference in color between the Control and CP pasta (3.5 < ΔE). This can be supported as the L* value for the Control was significantly higher (*p* < 0.05) than the cooked CP pasta. No differences were found for the a* and b* values between the cooked Control and CP pasta (*p* > 0.05).

There was no noticeable color difference between the dried pasta samples. However, once cooked, there was a clear color difference as the cooked Control pasta was found to be lighter than the CP pasta (*p* < 0.05). However, Duda et al. and Jakab et al. [50,80] found that lightness decreased while red and blue coloring increased with cricket powder replacement in pasta. For testing purposes, the caramel coloring was used to reduce differences in color between the CP pasta and Control. Though, concerns with caramel coloring may be that it may attribute a bitter taste in the pasta [81]. However, the appearance of insect-based pasta has been noted to be similar to whole wheat pasta which is considered healthier by consumers [50,73,80]. As a result, the darker coloring attributed to cricket powder may further support insect-based pasta to be perceived as a healthy alternative for consumers.

#### 4.2.3. Cooking and Textural Properties

There were no differences in OCT, water absorption, and cooking loss (*p* > 0.05). Both pasta samples had an OCT of approximately 5 min and 20 s. For textural properties, no differences were found for firmness, cohesiveness, and springiness between the Control and CP pasta (*p* > 0.05).

For OCT, no differences were found between the pasta versions (*p* > 0.05). However, previous studies have found that cooking time increased in insect-based pasta [50,73,80]. Though, Biró et al. [55] found that OCT decreased with silkworm powder in buckwheat-based pasta. Water absorption was slightly higher in the CP pasta than in the Control, though no difference (*p* > 0.05). Pasini et al. [80] observed an increase in WA with cricket (*Acheta domestica*) proteins, and Biró et al. [55] found that silkworm powder in pasta increased WA as well. This differs from Çabuk and Yılmaz [72] who found that WA decreased in pasta enriched with grasshopper or mealworm flour. However, this may reflect the effect of different insect species may have on the WA of pasta. 

The protein, dietary fiber, and starch content can influence the cooking properties of pasta [82]. Animal proteins can form gels which may increase water absorption [55,80,83]. However, WA can be an indicator of starch gelatinization [72,84]. The reduction of WA in previous studies may be a result of replacing wheat flour with insect powder. This would, in turn, reduce starch content in the pasta leading to a reduction in starch gelatinization. Cooking loss can also be used as an indicator for the quality of pasta regarding its overall cooking performance and resistance to disintegration during cooking [85]. When replacing durum wheat with another ingredient, the cooking loss can be a result of the pasta structure and the ingredient’s effect on gluten formation [55,72]. Plant-protein enriched pasta has been found to increase cooking loss [78,86]. However, previous research found that cooking loss decreased as cricket powder increased [50]. This may imply that cricket powder may be a viable option to increase the quality of enriched pasta. 

For textural properties, no differences were found for firmness, cohesiveness, and springiness *(p* > 0.05). Previous research has found that firmness increased in pasta containing insects which may be due to an increase in protein content [50]. There is still limited research on the use of commercial cricket powder in pasta. However, insect-enriched pasta may be a method to introduce alternative sources of protein into the consumer market. Pasta and other noodle products are ingrained into many cultures. Pasta may be perceived to be a more appropriate food product to contain insects than other staple foods such as bread [42,87,88]. Therefore, cricket powder or other insect powders may be a viable as an ingredient to improve the nutritional content and quality of those products. 

### 4.3. Brownies

**Figure 3:**
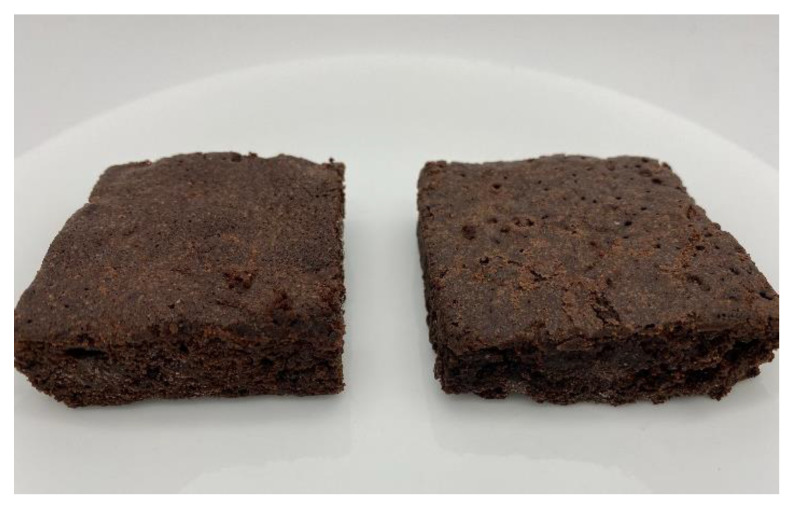
Control (**left**) and CP (**right**) brownies.

Figure 3 shows both versions of the brownies developed. The measurements conducted on the brownies are found in Table 6.

#### 4.3.1. Brownie Composition

Protein and fat content were found to be significantly higher (*p* < 0.05) in the CP brownie (19.26% and 38.95%, respectively) than in the Control (11.91% and 37.28%, respectively). The carbohydrate content was significantly lower (*p* < 0.05) in the CP brownie (46.93%) than in the Control (53.23%). No differences were found for moisture content and ash between the Control and CP brownies (*p* > 0.05).

The protein and fat content increased with a decrease in carbohydrates in the CP brownie when compared to the Control (*p* < 0.05). However, this may be attributed to the cricket powder which has a higher protein and fat content than wheat flour [64,71]. Previous research on other bakery products found that carbohydrate content decreased as protein and fat increased with the addition of insect flours [89,90,91,92]. Not to mention, the CP brownie had a slightly higher moisture content, though no difference (*p* > 0.05), than the Control which may be attributed to the fiber and protein from the cricket powder which retains more moisture [90,91]. As a result, this suggests that cricket powder can be used to improve the nutritional content of certain bakery products.

#### 4.3.2. Textural Properties

For textural properties, no differences were found for firmness and springiness when comparing both versions (*p* > 0.05). However, chewiness was significantly higher (*p* < 0.05) for the Control than the CP brownie.

The chewiness of the Control brownies was higher (*p* < 0.05) than the CP brownies, but no differences were found for firmness and cohesiveness (*p* > 0.05). This may be attributed to the low level of wheat flour in the CP brownie. The cricket powder may behave similarly to wheat bran by causing gluten dilution and limiting gluten formation in the product [93]. The lack of a gluten network may have reduced the viscoelasticity of the brownie preventing the expansion of gas cells that affect the textural properties. Previous research has shown that firmness and other textural parameters were reduced when wheat flour was replaced with insects in other bakery products [90,91,94,95]. De Oliveira et al. [92] found that hardness increased in bread when wheat flour was replaced with cockroach (*Nauphoeta cinerea*) flour; however, this may be due to the reduced air cell expansion in the bread from limited gluten formation. 

When replacing flour in bakery products, the physical qualities of the product may be altered due to the lack of gluten. Other studies have found that the use of pulse flours can improve the nutritional content but decrease the textural parameters, such as hardness, in sweet bakery products [96]. This relates to the amount of pulse flour replacement and the legume used and how it was processed (particle size). During preliminary testing, the particle size of the cricket powder was a concern as the brownies were found to have a grainy texture. As a result, the cricket powder was further ground. Chitin was found to have no emulsion capacity in wheat flour which may interfere in dough development [93]. Yeom et al. [97] found that rice bran dietary fiber increased hardness but led to a decrease in overall consumer acceptability and off-flavor characteristics. As a result, the amount of cricket powder and how it is processed may need to be a consideration when used in brownie formulations. Gurdian et al. [46] found that partial replacement of brownie mix with cricket powder decreased overall consumer acceptability. However, cricket powder may be a viable ingredient in brownies or similar bakery products. Consumers noted that the quality and overall acceptability of cookies containing cricket powder as acceptable as other cookies [98,99]. To the authors’ knowledge, this was the first study conducted on the effects of food quality in chocolate brownies enriched with cricket powder.

## 5. Conclusions

This preliminary study focused on developing three different products with partial replacement with cricket powder. Overall, the nutritional quality of the products was found to have increased with limited changes in quality parameters measured. As a result, the products developed may be comparable to conventional products already offered in the market. These results show the viability of partial cricket powder replacement in food products as well as supporting previous findings. In the case of sausage, this may be beneficial as it can reduce lean meat production while also maintaining the protein content of sausage products. For pasta, cricket powder may be a viable option to improve the nutritional profile of staple foods. Though brownies may be considered a high-calorie food product, the increase in protein may be attractive to certain consumers. Limitations to this study may be further analyses of each product such as consumer testing. However, the products developed would be used to predict the consumer acceptability of insect-based food products.

## Figures and Tables

**Table 1:** The usage levels of the ingredients used for the sausage blend with NO cricket powder (Control) and sausage blend containing cricket powder (CP). All percentages were rounded to the nearest hundredth place.

Ingredients	Control Usage Level (%)	CP Usage Level (%)	Ingredient Source & Location
Pork Shoulder Lean	62.29	56.06	Swift Meats, Greeley, CO, USA
Pork Shoulder Fat	31.15	31.15	Swift Meats, Greeley, CO, USA
Cricket Powder	-	6.23	JR Unique Foods, Udon Thani, Thailand
Fennel Seed, Ground	0.91	0.91	The Spice Hunter Inc., San Luis Obispo, CA, USA
Dried Basil	0.13	0.13	The Spice Hunter Inc., San Luis Obispo, CA, USA
Dried Oregano	0.16	0.16	Spicely Organics, Fremont, CA, USA
Crushed Red Chili Pepper	0.17	0.17	The Spice Hunter Inc., San Luis Obispo, CA, USA
Garlic, Minced	3.74	3.74	The Garlic Company, Bakersfield, CA, USA
Onion Powder	0.37	0.37	McCormick & Co., Inc., Hunt Valley, MD, USA
Black Pepper, Ground	0.09	0.09	McCormick & Co., Inc., Hunt Valley, MD, USA
Salt	0.76	0.76	First Street, Smart & Final, Commerce, CA, USA
Dried Thyme	0.14	0.14	The Spice Hunter Inc., San Luis Obispo, CA, USA
Dried Rosemary	0.10	0.10	Morton & Bassett Spices, Rohnert, CA, USA

**Table 2:** The usage levels of the ingredients used for the pasta with NO cricket powder (Control) and pasta containing cricket powder (CP). All percentages (except caramel coloring) were rounded to the nearest hundredth place.

Ingredients	Control Usage Level (%)	CP Usage Level (%)	Source & Location
Durum Wheat Semolina	61.41	73.87	Miller Milling Company, Fresno, CA, USA
Whole Wheat Flour	14.41	-	King Arthur Flour, Norwich, VT, USA
Cricket Powder	-	5.00	JR Unique Foods, Udon Thani, Thailand
P600 Powdered Caramel Coloring	0.10	0.075	Sethness Products Company, Clinton, IA, USA
Water	24.08	21.05	-

**Table 3:** The usage levels of the ingredients used for the brownies with NO cricket powder (Control) and brownies containing cricket powder (CP). All percentages were rounded to the nearest hundredth place.

Ingredient	Control Usage Level (%)	CP Usage Level (%)	Source & Location
Dutch Cocoa Powder	4.91	4.91	The Hershey Co., Hershey, PA, USA
Bittersweet Chocolate	11.86	11.86	Puratos Chocolate USA, Kenosha, WI, USA
Unsalted Butter	26.18	26.18	The Kroger Co., Cincinnati, OH, USA
Eggs, Beaten	16.36	16.36	The Kroger Co., Cincinnati, OH, USA
Granulated Sugar	21.27	21.27	Sysco Corp., Houston, TX, USA
Salt	0.36	0.36	First Street, Smart & Final, Commerce, CA, USA
Vanilla Extract	0.57	0.57	Cook Flavoring Co., Paso Robles, CA, USA
Bread Flour	9.41	2.41	General Mills Operations Inc., Minneapolis, MN, USA
Cricket Powder	-	7.00	JR Unique Foods, Udon Thani, Thailand
Bittersweet Chocolate Chunks	9.07	9.07	Puratos Chocolate USA, Kenosha, WI, USA

**Table 4:** Proximate composition of the raw sausage (on a dry basis), color, pH, cooking yield, and textural properties of both sausages developed. The means with 95% confidence intervals are shown. All percentages were rounded to the nearest hundredth place. Parameters that were denoted were found to be significantly different (*p* < 0.05).

Product Type	Control	CP
Proximate Composition		
Moisture (%) ^1^	59.77 ± 0.47	54.88 ± 1.19
Protein (%)	33.02 ± 1.06	32.38 ± 2.15
Fat (%) ^1^	48.61 ± 1.75	42.52 ± 1.47
Carbohydrates (%) ^1^	14.20 ± 0.16	20.81 ± 3.39
Ash (%)	4.17 ± 0.09	4.29 ± 0.09
Color (Raw)		
L*	31.10 ± 3.26	29.97 ± 1.12
a* ^1^	−1.44 ± 0.24	−2.42 ± 0.99
b*	8.53 ± 0.60	8.36 ± 0.94
ΔE	-	1.50
Color (Cooked)		
L*	30.86 ± 1.35	30.05 ± 1.44
a* ^1^	−1.02 ± 0.79	−2.08 ± 0.39
b* ^1^	10.25 ± 0.79	8.98 ± 0.42
ΔE	-	1.84
pH		
Raw	6.20 ± 0.07	6.18 ± 0.03
Cooked	6.36 ± 0.08	6.34 ± 0.17
Cooking Yield (%) ^1^	69.08 ± 4.05	81.08 ± 3.55
Textural Properties		
Firmness (N) ^1^	23.78 ± 2.01	16.35 ± 2.95
Cohesiveness ^1^	0.45 ± 0.07	0.34 ± 0.09
Springiness (mm) ^1^	6.85 ± 0.86	8.71 ± 1.43

^1^ A significant difference was found between the Control and CP versions using a two-sample *t*-Test (*p* < 0.05).

**Table 5:** Proximate composition of the dried pasta (on a dry basis), color, cooking properties, and textural properties of both dried pastas developed. The means with 95% confidence intervals are shown. All percentages were rounded to the nearest hundredth place. Parameters that were denoted were found to be significantly different (*p* < 0.05).

Product Type	Control	CP
Proximate Composition		
Moisture (%)	5.62 ± 0.55	5.87 ± 0.34
Protein (%) ^1^	13.13 ± 3.16	16.83 ± 0.24
Fat (%) ^1^	0.22 ± 0.36	1.23 ± 0.17
Carbohydrates (%) ^1^	82.48 ± 3.50	77.65 ± 0.23
Ash (%)	1.02 ± 0.01	1.04 ± 0.14
Color (Dried)		
L*	30.71 ± 1.08	30.66 ± 1.07
a*	−1.82 ± 0.49	−1.74 ± 0.49
b*	8.93 ± 1.02	9.04 ± 0.19
ΔE	-	0.15
Color (Cooked)		
L* ^1^	58.16 ± 2.51	54.67 ± 4.34
a*	1.34 ± 0.89	0.91 ± 0.83
b*	13.47 ± 2.86	12.58 ± 2.21
ΔE	-	3.63
Cooking Properties		
OCT (min)	5.36 ± 0.44	5.28 ± 0.44
WA (%)	140.33 ± 10.25	149.33 ± 10.25
Cooking Loss (%)	6.18 ± 2.89	6.06 ± 0.97
Textural Properties		
Firmness (N)	1.84 ± 0.49	1.92 ± 0.10
Cohesiveness	0.40 ± 0.48	0.23 ± 0.29
Springiness (mm)	0.50 ± 0.19	0.50 ± 0.29

^1^ A significant difference was found between the Control and CP versions using a two-sample *t*-Test (*p* < 0.05).

**Table 6:** Proximate composition of the baked brownie (on a dry basis) and textural properties of both brownies developed. The means with 95% confidence intervals are shown. All percentages were rounded to the nearest hundredth place. Parameters that were denoted were found to be significantly different (*p* < 0.05).

Product Type	Control	CP
Proximate Composition		
Moisture (%)	11.47 ± 2.41	11.94 ± 2.41
Protein (%) ^1^	11.91 ± 0.55	19.26 ± 1.99
Fat (%) ^1^	37.28 ± 0.85	38.95 ± 0.86
Carbohydrates (%) ^1^	53.23 ± 0.90	46.93 ± 1.68
Ash (%)	2.03 ± 0.26	2.08 ± 0.26
Textural Properties		
Firmness (N)	38.44 ± 13.22	37.01 ± 7.86
Springiness (mm)	7.67 ± 2.94	6.03 ± 1.56
Chewiness (mJ) ^1^	81.47 ± 34.54	38.33 ± 16.38

^1^ A significant difference was found between the Control and CP versions using a two-sample *t*-Test (*p* < 0.05).

## Data Availability

The data presented in this study are available on request from the corresponding author. The data are not publicly available due to privacy restrictions.

## Appendix A

**Table A1:** Nutritional data of the cricket powder. This information is based on 100 g of powder based on the item description/nutrition facts from the JR Unique Foods website [100].

Nutrient	Amount per 100 g
Protein	78.0 g
Calories from Fat	164 calories
Calories	410 calories
Saturated Fat	2.2 g
Total Fat	6.7 g
Trans-fatty Acid	0.02 g
Carbohydrate	5.5 g
Calcium	125 mg
Potassium	1.304 mg
Fiber	5.4 g
Total sugar	0.2 g
Vitamin B12	5.5 g
Iron	16.6 mg
Cholesterol	6.67 mg
Ash	4.85 g
Choline	189 mg
Folic Acid	218 mg
